# Longitudinal outcomes associated with structured exercise and dietary and microbiota-targeted care in patients with diabetic sarcopenia: a retrospective cohort study

**DOI:** 10.3389/fendo.2026.1923121

**Published:** 2026-09-22

**Authors:** Xiaowei Huang, Jingyu Duan, Xue Liang, Yuzheng Li, Yongcai Zhao

**Affiliations:** 1 Department of Endocrinology, Cangzhou Central Hospital, Cangzhou, Hebei, China; 2 Department of Ultrasonography, Cangzhou Central Hospital, Cangzhou, Hebei, China

**Keywords:** diabetic sarcopenia, dietary counseling, microbiota-targeted care, structured exercise, type 2 diabetes mellitus

## Abstract

**Background:**

Diabetic sarcopenia is associated with functional impairment and metabolic abnormalities in patients with type 2 diabetes mellitus (T2DM). Real-world evidence regarding structured exercise combined with dietary and microbiota-targeted care remains limited.

**Methods:**

This retrospective cohort study included 128 patients with T2DM and sarcopenia between January 2022 and December 2024. Based on the treatment received, patients were divided into a control group receiving conventional diabetes management (n = 63) and an intervention group receiving conventional management combined with structured exercise, dietary and microbiota-targeted care (n = 65). Outcomes were assessed at baseline and months 1, 3, 6, 9, and 12. The primary outcome was the longitudinal change in skeletal muscle index (SMI). Secondary outcomes included handgrip strength, gait speed, Short Physical Performance Battery (SPPB) score, glycated hemoglobin A1c (HbA1c), fasting plasma glucose (FPG), and homeostasis model assessment of insulin resistance (HOMA-IR), as well as the incidence of adverse events. Adjusted generalized estimating equation models were used to evaluate longitudinal trajectories.

**Results:**

Significant group × time interactions were observed for SMI (P = 0.017), handgrip strength (P < 0.001), gait speed (P < 0.001), and SPPB score (P = 0.015). At month 12, adjusted between-group differences favoring the intervention group were 0.282 kg/m² for SMI (95% CI, 0.177–0.387), 3.348 kg for handgrip strength (95% CI, 2.718–3.983), 0.129 m/s for gait speed (95% CI, 0.109–0.149), and 1.225 points for SPPB score (95% CI, 0.895–1.556; all P < 0.001). Group × time interactions were not significant for HbA1c, HOMA-IR, or FPG. No significant difference in adverse-event incidence was detected (Risk ratio: 1.171; P = 0.478).

**Conclusions:**

Structured exercise combined with dietary and microbiota-targeted care was associated with more favorable muscle-related trajectories but not superior overall glycemic control. The observational design precludes causal inference or separation of individual component effects, warranting confirmation in prospective randomized trials.

## 1 Introduction

Diabetic sarcopenia is a diabetes-associated complication characterized by progressive loss of skeletal muscle mass, muscle strength, and physical function in individuals with type 2 diabetes mellitus (T2DM) (1). With the increasing prevalence of T2DM and population aging, its burden is rapidly growing (2, 3). Patients with T2DM are at substantially higher risk of sarcopenia, which is associated with functional decline, falls, hospitalization, reduced quality of life, and increased mortality (4). Beyond classical diabetic complications, diabetic sarcopenia is a serious and underrecognized condition, underscoring an urgent need for effective management strategies (5).

Current conventional management of T2DM primarily focuses on glycemic control and cardiovascular risk reduction, which are essential for preventing classical diabetic complications (6). However, these strategies are not specifically designed to preserve skeletal muscle mass, muscle strength, or physical performance, which are the core features of sarcopenia (7). As a result, muscle-related outcomes may receive limited attention in routine diabetes care, and sarcopenia may progress despite adequate glycemic management (8, 9). Although general advice regarding nutrition and physical activity is commonly provided, it may not be sufficiently structured or individualized to counteract age- and diabetes-related muscle catabolism (10). In addition, some antidiabetic medications may have neutral or potentially unfavorable effects on muscle metabolism, particularly in older adults with long-standing disease (11). These limitations highlight the need for complementary strategies that directly address musculoskeletal health in patients with T2DM.

The pathophysiology of diabetic sarcopenia is multifactorial, involving insulin resistance, chronic inflammation, mitochondrial dysfunction, and impaired muscle protein synthesis (12). In addition to metabolic dysregulation, the gut microbiota has recently emerged as a potential contributor to host metabolism and skeletal muscle health (13, 14). Alterations in gut microbial composition and function have been linked to insulin resistance, systemic inflammation, and metabolic endotoxemia, all of which may contribute to muscle wasting (15). Emerging evidence suggests that reductions in beneficial microbial metabolites, such as short-chain fatty acids, may impair muscle energy metabolism and anabolic responsiveness (16–18). These findings support the concept of a gut–muscle axis and suggest that microbiota-targeted strategies may warrant further investigation as complementary approaches to conventional diabetes treatment.

Exercise intervention is a cornerstone non-pharmacological strategy for preventing and managing sarcopenia, with resistance training shown to improve muscle mass, strength, physical performance, and insulin sensitivity in patients with T2DM (19). However, in real-world clinical settings, exercise interventions are often underutilized or insufficiently structured, and their effects may be attenuated in individuals with advanced metabolic dysfunction or persistent inflammatory burden (20, 21). Consequently, combined approaches integrating exercise with dietary and microbiota-targeted care have attracted increasing interest (22). Despite increasing mechanistic insights, clinical evidence evaluating structured exercise combined with dietary counseling and microbiota-targeted supplementation in patients with diabetic sarcopenia remains limited. Most existing studies have focused on single interventions and are often conducted in controlled experimental settings with short follow-up durations (23, 24). In particular, real-world data comparing longitudinal muscle-related, metabolic, and safety outcomes associated with combined care and conventional diabetes management remain scarce.

Therefore, this retrospective cohort study aimed to examine the association of a combined care package comprising structured exercise, dietary counseling, and optional microbiota-targeted supplementation with longitudinal muscle, physical performance, metabolic, and safety outcomes in patients with diabetic sarcopenia.

## 2 Materials and methods

### 2.1 Study design and participants

This retrospective cohort study was conducted at the Department of Endocrinology, Cangzhou Central Hospital between January 2022 and December 2024. A total of 283 patients with T2DM and sarcopenia were identified from the electronic medical records. Of these, 51 were excluded according to the clinical eligibility criteria, leaving 232 eligible patients. Patients were classified according to treatment initiated: 106 received conventional management and 126 received combined care. Further exclusions were due to incomplete follow-up, missing data, or inadequate adherence to the additional 8-week intervention. Consequently, 128 patients with complete data at baseline and months 1, 3, 6, 9, and 12 were included. Because inclusion in the combined-intervention group also required adequate post-initiation adherence, this was an adherent complete-case analysis rather than an analysis of all treatment initiators. The final analytical cohort comprised 63 patients in the control group who received conventional diabetes management alone and 65 patients in the intervention group who received conventional diabetes management combined with structured exercise, dietary counseling, and microbiota-targeted care. The complete participant-selection process is shown in **Figure 1**. Treatment strategies were determined based on baseline physical function, comorbidities, exercise tolerance, dietary habits, gastrointestinal tolerance, and the treating physician’s clinical judgment. T2DM was diagnosed according to established international guidelines, and sarcopenia was defined according to the Asian Working Group for Sarcopenia (AWGS) 2019 criteria (25). The study protocol was approved by the Institutional Ethics Committee of the Cangzhou Central Hospital (Approval No. 2025-374-02; approval date: November 3, 2025). As this study was retrospective and based on anonymized clinical data, the requirement for written informed consent was waived.

**Figure 1:**
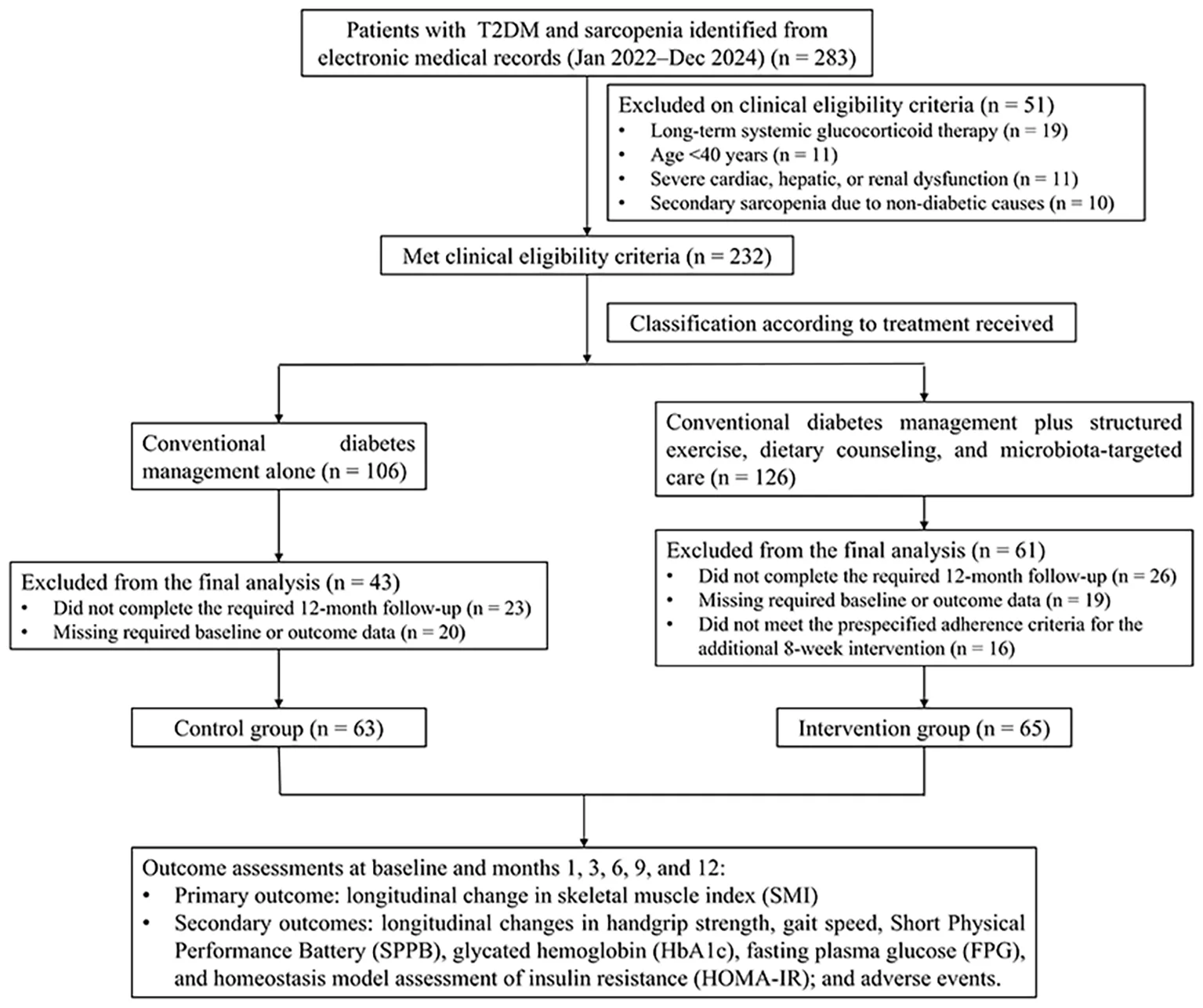
The flow chart of this study. Patients were classified at the start of follow-up according to the treatment initiated. Exclusion categories were mutually exclusive, and each patient was counted once according to the primary documented reason for exclusion. The adherence criteria applied only to the additional 8-week structured exercise, dietary counseling, and, where prescribed, microbiota-targeted supplementation components, rather than to conventional diabetes management. Although the records identified 16 patients who did not meet the prespecified adherence criteria, the specific reasons for inadequate adherence, discontinuation, or treatment modification were not consistently documented. All 128 patients included in the final analysis had complete data at baseline and months 1, 3, 6, 9, and 12.

Inclusion criteria: (1) diagnosis of T2DM; (2) a diagnosis of sarcopenia based on the AWGS 2019 criteria; (3) age ≥ 40 years; and (4) availability of complete baseline clinical data and follow-up assessments for at least 12 months.

Exclusion criteria: (1) acute diabetic complications (e.g., diabetic ketoacidosis or hyperosmolar hyperglycemic state) within 3 months before enrollment; (2) severe cardiac, hepatic, or renal dysfunction that could substantially limit participation in physical activity; (3) secondary sarcopenia due to non-diabetic causes or conditions known to cause significant muscle wasting, such as active malignancy, neuromuscular disorders, chronic inflammatory or autoimmune diseases, advanced organ failure, or endocrine disorders affecting muscle metabolism; (4) long-term systemic glucocorticoid therapy; and (5) incomplete clinical records or missing key outcome data.

### 2.2 Conventional diabetes management

All patients in both groups received standard diabetes care in accordance with contemporary clinical guidelines. Conventional management focused primarily on glycemic control and included individualized pharmacological therapy, routine lifestyle counseling, and management of cardiovascular risk factors. Antidiabetic medications, including oral hypoglycemic agents and/or insulin, were prescribed and adjusted by treating physicians based on glycemic targets, comorbidities, and patient tolerance. Routine lifestyle counseling consisted of general advice regarding healthy diet, physical activity, and weight management, delivered as part of usual clinical care. Patients in the control group did not receive a structured exercise prescription or targeted gut microbiota regulation beyond routine clinical advice.

### 2.3 Exercise combined with dietary and microbiota-targeted care

Patients in the intervention group participated in a structured and supervised exercise program primarily focused on resistance training, supplemented by aerobic and balance exercises. The exercise program was conducted at our hospital-based rehabilitation center. Exercise prescriptions were developed by rehabilitation therapists or trained clinicians based on baseline physical function, comorbid conditions, and exercise tolerance, and were adjusted during the 8-week program as needed. Each training session lasted approximately 40–60 minutes and was performed 2–3 times per week for 8 consecutive weeks. Resistance training constituted the core component of the program and targeted major muscle groups of the upper and lower extremities, including the quadriceps, hamstrings, gluteal muscles, calf muscles, and major upper-limb muscle groups. Typical resistance exercises included multi-joint and single-joint movements using body weight, elastic bands, or resistance machines, depending on individual capability and safety considerations. Each session included approximately six to eight resistance exercises. Participants initially completed two sets of 12–15 repetitions and progressed to two or three sets of 8–12 repetitions, with approximately 60–90 s of rest between sets. Each repetition was performed at a controlled pace, with approximately 2 s for the concentric phase and 2 s for the eccentric phase, through the available range of motion. Exercise intensity for resistance training was prescribed at a moderate level, corresponding to approximately 50–70% of the estimated one-repetition maximum (1RM) and a score of 12–14 on the 6–20 Borg Rating of Perceived Exertion (RPE) scale. The 1RM was estimated from the maximum load that could be completed for approximately 8–10 repetitions with correct technique using the Epley equation: estimated 1RM = load × (1 + number of repetitions/30). RPE was assessed at the end of each exercise set and was used together with movement quality and exercise tolerance to adjust the training load. Intensity was standardized across body-weight, elastic-band, and machine-based exercises by selecting a resistance that allowed completion of the prescribed repetition range within the target RPE. When participants completed the upper limit of the prescribed repetition range with correct technique and an RPE below the target range during two consecutive sessions, the resistance was increased by approximately 5–10% or advanced to the next available elastic-band resistance level. Proximity to neuromuscular failure and repetitions in reserve were not routinely documented. Aerobic exercise, such as walking or cycling, was incorporated for 10–20 minutes per session at a moderate intensity corresponding to an RPE of approximately 11–13 to improve cardiometabolic fitness. The duration was generally initiated at approximately 10 minutes and gradually increased to 20 minutes according to individual exercise tolerance. Balance training, including static and dynamic balance exercises, was included to reduce fall risk and improve functional stability. Balance exercises were performed for approximately 5–10 minutes per session and included tandem standing, supported single-leg standing, and multidirectional stepping, as appropriate to the participant’s functional capacity.

Before participation, patients underwent routine clinical assessment of cardiovascular status, musculoskeletal limitations, diabetes-related complications, and exercise tolerance. Capillary blood glucose and symptoms of hypoglycemia were assessed before exercise and, when clinically indicated, during or after exercise. Exercise intensity was reduced, or the session was terminated, in the presence of symptomatic hypoglycemia, clinically significant hyperglycemia identified according to the departmental diabetes-exercise protocol, chest discomfort, dizziness, unusual dyspnea, abnormal blood pressure responses, or musculoskeletal pain. Hypoglycemic events were managed according to the hospital’s routine diabetes-care procedures.

Dietary and microbiota-targeted care consisted of structured dietary counseling and, when clinically indicated, microbiota-targeted supplementation. The intervention was delivered during the 8-week program by treating physicians. Dietary counseling focused on modifying dietary patterns potentially supportive of gut microbial health, with emphasis on increasing the intake of fiber-rich foods such as whole grains, vegetables, legumes, and fruits, while maintaining dietary balance appropriate for glycemic control. Dietary recommendations were individualized based on baseline dietary habits, gastrointestinal tolerance, and comorbid conditions, and were documented in routine clinical records. In addition to dietary counseling, probiotic, prebiotic, or synbiotic supplementation was prescribed at the discretion of the treating physician when deemed appropriate. The choice of supplementation was not standardized and reflected routine clinical practice rather than a fixed experimental protocol. The probiotic preparations most commonly contained viable *Bifidobacterium*, *Lactobacillus*, and *Enterococcus* species and were administered orally. Prebiotic supplementation consisted primarily of inulin or fructo-oligosaccharides at a total daily dose of approximately 5–10 g. Synbiotic preparations contained combinations of *Bifidobacterium* and/or *Lactobacillus* species with inulin or fructo-oligosaccharides and were generally administered once daily. When prescribed, supplementation was administered for 8 weeks. The supplementation categories were not mutually exclusive. Some patients received more than one type of supplementation. All 65 patients received structured dietary counseling. Among them, 30 received probiotics, 18 received prebiotics, and 22 received synbiotics, while 12 received no supplementation. The intervention details and numbers of recipients are summarized in **Supplementary Table S1**.

Adherence during the 8-week combined intervention was assessed retrospectively using exercise attendance records, exercise logs, prescription records, dietary counseling records, and follow-up notes. Exercise and supplementation adherence were calculated as the proportions of prescribed sessions completed and prescribed days of use documented, respectively. Dietary adherence was calculated as the proportion of scheduled assessments documenting compliance with dietary recommendations; no validated quantitative dietary instrument was used. A prespecified threshold of ≥80% was used as a pragmatic operational criterion for adequate exposure to each applicable component rather than as a biologically validated cutoff. The supplementation criterion applied only to patients prescribed supplementation. Only patients meeting the applicable exercise, dietary, and supplementation adherence criteria were included in the intervention-group analysis. Specific reasons for inadequate adherence or treatment discontinuation were not consistently documented and could not be reliably categorized.

After the 8-week intervention, supervised exercise and prescribed microbiota-targeted supplementation were discontinued. Patients then were encouraged to continue home-based exercise and the recommended dietary pattern. No standardized supervised maintenance program was implemented.

### 2.4 Baseline data collection

Baseline demographic, clinical, metabolic, and functional data were extracted retrospectively from electronic medical records at the time of study entry. Demographic variables included age and sex. Anthropometric data included body mass index (BMI, kg/m²). Clinical characteristics included duration of T2DM, use of insulin therapy, and history of antibiotic exposure within the preceding 3 months. Baseline glycemic control was assessed using glycated hemoglobin A1c (HbA1c, %) and fasting plasma glucose (FPG, mmol/L). Insulin resistance was estimated using the homeostasis model assessment of insulin resistance (HOMA-IR). Skeletal muscle mass was evaluated using the skeletal muscle index (SMI, kg/m²). Muscle strength was assessed by handgrip strength (kg), and physical performance was evaluated using usual gait speed (m/s) and the Short Physical Performance Battery (SPPB) score.

### 2.5 Outcome measures

Outcome measures were assessed at baseline and at 1, 3, 6, 9, and 12 months. The primary outcome was the longitudinal change in SMI over the 12-month follow-up period. Secondary outcomes were longitudinal changes in handgrip strength, gait speed, SPPB score, HbA1c, FPG, and HOMA-IR over the follow-up period. Adverse events recorded during the 12-month follow-up were also evaluated as a secondary safety outcome.

SMI was calculated as appendicular lean mass divided by height squared (kg/m²). Appendicular lean mass was measured by multifrequency segmental bioelectrical impedance analysis using an InBody S10 body composition analyzer (InBody Co., Ltd., Seoul, Republic of Korea). Measurements were performed in the morning after an overnight fast, with participants in the supine position. Appendicular lean mass was calculated as the sum of the lean mass of both arms and both legs. Height was measured without shoes to the nearest 0.1 cm using an automatic height and weight scale (HW-700, Henan Lejia Electronic Technology Co., Ltd., Zhengzhou, China). The same equipment models and standardized procedures were used throughout the study period, and baseline height was used to calculate SMI at all assessment time points. According to the AWGS criteria, low muscle mass is defined as an SMI < 7.0 kg/m² in men and < 5.7 kg/m² in women (25).

Muscle strength was assessed using an EH101 electronic hand dynamometer (CAMRY, Guangdong, China), with the result recorded in kilograms. Participants were seated with the elbow flexed at 90° and the forearm in a neutral position and were instructed to squeeze the dynamometer as forcefully as possible for approximately 3–5 s. The dominant hand was assessed unless pain, injury, or another clinical limitation prevented its use, in which case the nondominant hand was tested. Three maximal voluntary contractions were performed with a brief rest between trials, and the highest recorded value was used for analysis. The same hand and standardized measurement procedure were used at all available follow-up assessments whenever possible. According to the AWGS criteria, low muscle strength is defined as handgrip strength < 28 kg in men and < 18 kg in women (25).

Physical performance was evaluated using gait speed and the SPPB. Gait speed was measured over a clearly marked 6-m course on a flat, unobstructed surface according to the routine departmental protocol. Participants were instructed to walk at their usual pace, and timing began when the first foot crossed the starting line and ended when the first foot crossed the finish line. Two trials were performed with a brief rest between trials, and the faster gait speed, expressed in meters per second (m/s), was used for analysis. Usual walking aids were permitted when necessary and were kept consistent across follow-up assessments whenever possible. Reduced physical performance is commonly defined as a gait speed <1.0 m/s.

The SPPB consists of three components: balance tests, a 4-m walking speed test, and a repeated chair stand test (26). For the balance assessment, participants maintained side-by-side, semi-tandem, and tandem positions for up to 10 s each. The faster of two trials was used for the 4-m walking test. The chair stand test was performed using a standard armless chair, with participants instructed to complete five consecutive stands as quickly as possible while keeping their arms folded across their chest. Each component is scored from 0 to 4, yielding a total score ranging from 0 to 12, with lower scores indicating poorer physical performance and greater risk of disability.

Metabolic outcomes included HbA1c, FPG, and the HOMA-IR. Fasting venous blood samples were collected in the morning after an overnight fast of at least 8 h. FPG was measured using the hexokinase method with an AU5800 automated chemistry analyzer (Beckman Coulter, USA), HbA1c by high-performance liquid chromatography using an HLC-723G8 analyzer (Tosoh Corporation, Japan), and fasting serum insulin by electrochemiluminescence immunoassay using a cobas e601 analyzer (Roche Diagnostics, Germany). HOMA-IR was calculated as FPG (mmol/L) × fasting serum insulin (μU/mL)/22.5. HOMA-IR was calculated to estimate insulin resistance, with higher values indicating greater metabolic impairment.

Body-composition and physical-function assessments were conducted as part of routine clinical care by trained nurses or technicians using standardized departmental procedures. The equipment was routinely inspected and calibrated or verified in accordance with the manufacturers’ instructions.

Adverse events occurring during the follow-up were reviewed retrospectively from electronic medical records and follow-up documentation.

### 2.6 Statistical analysis

Statistical analyses were performed using SPSS version 26.0 (IBM Corp., Armonk, NY, USA) and R version 4.2.3 (R Foundation for Statistical Computing, Vienna, Austria). Continuous variables were first assessed for normality using the Shapiro–Wilk test. Normally distributed data were presented as mean ± standard deviation (SD). Baseline characteristics were assessed using standardized mean differences (SMDs), with an absolute SMD <0.10 indicating negligible imbalance. Categorical variables were presented as numbers and percentages (%) and were compared using the chi-square test or Fisher’s exact test, as appropriate. All 128 patients included in the final analytical cohort had complete measurements for every outcome at baseline and months 1, 3, 6, 9, and 12. Therefore, no outcome data were missing within the analytical cohort, and no missing-data imputation was performed. Generalized estimating equation (GEE) models were used to examine longitudinal outcome trajectories and account for within-participant correlations. GEE models were fitted using the geepack package (version 1.3.13). For each outcome, measurements obtained at months 1, 3, 6, 9, and 12 were modeled as repeated dependent observations. Each model included group, time, and the group × time interaction. Each model was adjusted for the corresponding baseline outcome value, age, sex, BMI, duration of diabetes, insulin use, and antibiotic exposure. Time was treated as a categorical variable, with month 1 as the reference category. The overall group × time interaction was used to determine whether longitudinal trajectories differed between the intervention and control groups. Adjusted between-group differences at month 12 were estimated from the GEE models with 95% confidence intervals (CIs). The overall group × time interaction for SMI was the primary longitudinal test. The adjusted between-group difference in SMI at month 12 was designated as the primary time-specific contrast; the remaining outcome comparisons were secondary. An autoregressive working correlation structure (AR(1)) was specified to account for correlations among repeated measurements within participants. Robust sandwich standard errors were used for statistical inference. Gaussian GEE models with identity links were used for all continuous outcomes. Outcome distributions and model residuals were examined for marked non-normality, heteroscedasticity, and influential observations. Although SPPB scores are bounded from 0 to 12, the observed scores ranged from 5 to 11 without accumulation at the scale boundaries. Therefore, the Gaussian GEE model with robust standard errors was retained for SPPB. Model-fit indices were reported using the quasi-likelihood under the independence model criterion (QIC) and corrected QIC (QICC). All statistical tests were two-sided, and a p-value < 0.05 was considered statistically significant.

## 3 Results

### 3.1 Baseline characteristics

A total of 128 patients were included, with 63 in the control group and 65 in the intervention group (**Table 1**). Relatively small between-group differences were observed for SMI (absolute SMD = 0.076), handgrip strength (0.058), HbA1c (0.078), FPG (0.087), and antibiotic exposure (0.054). The largest baseline differences were observed for BMI (0.303) and gait speed (0.275). Differences were also observed for diabetes duration (0.185), insulin use (0.154), HOMA-IR (0.123), SPPB score (0.113), age (0.108), and sex (0.100).

**Table 1:** Baseline characteristics of the study participants.

Variables	Control group (n = 63)	Intervention group (n = 65)	Absolute SMD
Age (years)	66.35 ± 5.31	65.80 ± 4.85	0.108
Sex (male/female)	38 (60.3%)/25 (39.7%)	36 (55.4%)/29 (44.6%)	0.100
BMI (kg/m²)	22.54 ± 2.02	23.21 ± 2.36	0.303
Duration of diabetes (years)	13.48 ± 4.50	12.62 ± 4.80	0.185
HbA1c (%)	8.13 ± 0.97	8.06 ± 0.91	0.078
FPG (mmol/L)	7.83 ± 1.08	7.93 ± 1.25	0.087
HOMA-IR	3.82 ± 0.58	3.90 ± 0.64	0.123
SMI (kg/m²)	5.41 ± 0.51	5.37 ± 0.50	0.076
Handgrip strength (kg)	18.70 ± 3.61	18.92 ± 3.98	0.058
Gait speed (m/s)	0.89 ± 0.09	0.91 ± 0.06	0.275
SPPB score	7.11 ± 1.23	7.26 ± 1.42	0.113
Insulin use (yes/no)	28 (44.4%)/35 (55.6%)	24 (36.9%)/41 (63.1%)	0.154
Antibiotic exposure (yes/no)	14 (22.2%)/49 (77.8%)	13 (20.0%)/52 (80.0%)	0.054

Data are presented as mean ± standard deviation (SD) for continuous variables and number (%) for categorical variables. Between-group differences were assessed using standardized mean differences (SMDs), with an absolute SMD <0.10 indicating negligible imbalance. BMI, body mass index; FPG, fasting plasma glucose; SMI, skeletal muscle index; SPPB, Short Physical Performance Battery; HbA1c, glycated hemoglobin; HOMA-IR, homeostasis model assessment of insulin resistance.

### 3.2 Longitudinal changes in muscle mass and physical function

In the adjusted GEE models, significant group × time interactions were observed for the primary longitudinal outcome, SMI (Wald χ² = 12.100, P = 0.017), and for the secondary longitudinal outcomes of handgrip strength (Wald χ² = 61.900, P < 0.001), gait speed (Wald χ² = 142.000, P < 0.001), and SPPB score (Wald χ² = 12.425, P = 0.015). These findings indicated that the longitudinal trajectories of all four outcomes differed between the intervention and control groups. The observed longitudinal trajectories with 95% CIs are presented in **Figure 2**. With the control group and month 1 as the reference categories, significant group effects at month 1 were observed for SMI (Wald χ² = 4.988, P = 0.026), handgrip strength (Wald χ² = 11.260, P < 0.001), gait speed (Wald χ² = 67.800, P < 0.001), and SPPB score (Wald χ² = 13.400, P < 0.001). In the control group, the time effect was significant for gait speed (Wald χ² = 38.000, P < 0.001), but not for SMI (P = 0.131), handgrip strength (P = 0.992), or SPPB score (P = 0.245). The corresponding baseline outcome value was significantly associated with each longitudinal outcome (all P < 0.05). Age was associated with handgrip strength, gait speed, and SPPB score, but not with SMI. Sex, BMI, diabetes duration, insulin use, and antibiotic exposure were not significantly associated with these outcomes (**Table 2**). For the primary time-specific contrast, the intervention group had a significantly higher adjusted SMI than the control group at month 12 (adjusted between-group difference: 0.282 kg/m²; 95% CI: 0.177–0.387; P < 0.001). Significant adjusted month-12 differences favoring the intervention group were also observed for handgrip strength (adjusted between-group difference: 3.348 kg; 95% CI: 2.718–3.983), gait speed (adjusted between-group difference: 0.129 m/s; 95% CI: 0.109–0.149), and SPPB score (adjusted between-group difference: 1.225 points; 95% CI: 0.895–1.556; all P < 0.001) (**Table 3**).

**Figure 2:**
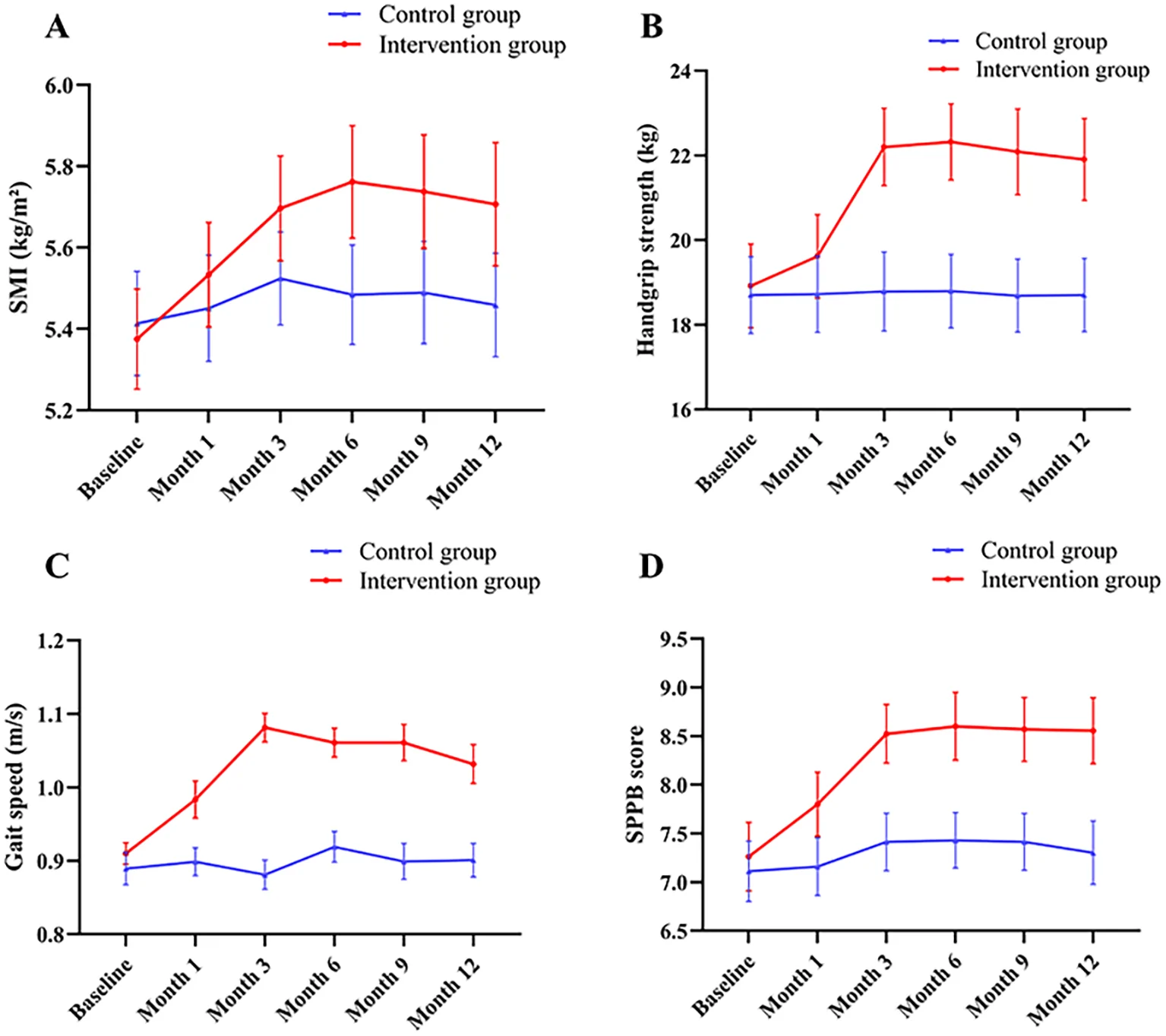
Longitudinal changes in SMI, handgrip strength, gait speed, and SPPB score. **(A)** SMI; **(B)** handgrip strength; **(C)** gait speed; and **(D)** SPPB score. Values are presented as observed means with 95% CI at baseline and months 1, 3, 6, 9, and 12. At each assessment, the control and intervention groups included 63 and 65 participants, respectively. Adjusted GEE analyses showed significant group × time interactions for SMI (P = 0.017), handgrip strength (P < 0.001), gait speed (P < 0.001), and SPPB score (P = 0.015). CI, confidence interval; GEE, generalized estimating equation; SMI, skeletal muscle index; SPPB, Short Physical Performance Battery.

**Table 2:** Longitudinal changes in SMI, handgrip strength, gait speed, and SPPB score.

Variable	SMI (kg/m²)			Handgrip strength (kg)			Gait speed (m/s)			SPPB score		
	Wald χ²	df	P	Wald χ²	df	P	Wald χ²	df	P	Wald χ²	df	P
Group	4.988	1	0.026	11.260	1	<0.001	67.800	1	<0.001	13.400	1	<0.001
Time	7.087	4	0.131	0.272	4	0.992	38.000	4	<0.001	5.439	4	0.245
Group × Time	12.100	4	0.017	61.900	4	<0.001	142.000	4	<0.001	12.425	4	0.015
Corresponding baseline outcome value	5.736	1	0.017	103.720	1	<0.001	49.200	1	<0.001	67.000	1	<0.001
Age	0.132	1	0.716	99.600	1	<0.001	62.800	1	<0.001	24.300	1	<0.001
Sex	0.437	1	0.509	0.056	1	0.814	0.797	1	0.372	0.051	1	0.821
BMI	2.753	1	0.097	2.052	1	0.152	2.443	1	0.118	1.608	1	0.205
Duration of diabetes	2.826	1	0.093	0.707	1	0.401	0.587	1	0.444	1.462	1	0.227
Insulin use	2.879	1	0.090	0.015	1	0.902	0.001	1	0.972	0.002	1	0.964
Antibiotic exposure	1.482	1	0.223	1.157	1	0.282	0.281	1	0.596	0.787	1	0.375

Values are Wald χ² statistics, degrees of freedom (df), and P values from GEE models. Measurements at months 1, 3, 6, 9, and 12 were modeled as repeated outcomes. Models included group, time, and group × time and were adjusted for the corresponding baseline outcome value, age, sex, BMI, diabetes duration, insulin use, and antibiotic exposure. An AR(1) correlation structure and robust sandwich standard errors were used. The group × time interaction represents differences in longitudinal trajectories between groups. Model-fit indices were: SMI, QIC/QICC = 114.277/120.553; handgrip strength, 2338.514/2344.789; gait speed, 62.729/69.004; and SPPB score, 594.498/600.773. SMI, skeletal muscle index; SPPB, Short Physical Performance Battery; QIC, quasi-likelihood under the independence model criterion; QICC, corrected QIC.

**Table 3:** GEE-derived adjusted between-group contrasts in SMI, handgrip strength, gait speed, SPPB score, HbA1c, HOMA-IR, and FPG at month 12.

Outcome	Control group (n = 63)	Intervention group (n = 65)	Adjusted between-group difference (95% CI)	P
SMI (kg/m²)	5.46 ± 0.51	5.71 ± 0.61	0.282 (0.177 to 0.387)	<0.001
Handgrip strength (kg)	18.70 ± 3.44	21.91 ± 3.89	3.348 (2.718 to 3.983)	<0.001
Gait speed (m/s)	0.90 ± 0.09	1.03 ± 0.11	0.129 (0.109 to 0.149)	<0.001
SPPB score (points)	7.30 ± 1.29	8.55 ± 1.37	1.225 (0.895 to 1.556)	<0.001
HbA1c (%)	7.52 ± 1.01	7.45 ± 0.92	0.013 (−0.183 to 0.209)	0.894
HOMA-IR	3.72 ± 0.65	3.52 ± 0.73	−0.204 (−0.371 to −0.038)	0.016
FPG (mmol/L)	7.28 ± 1.09	7.04 ± 0.99	−0.254 (−0.473 to −0.034)	0.024

Month-12 values are presented as unadjusted observed means ± standard deviations (SDs). Adjusted between-group differences and 95% confidence intervals (CIs) were estimated from GEE models and represent the intervention group minus the control group. Each model included post-baseline measurements at months 1, 3, 6, 9, and 12 and was adjusted for the corresponding baseline outcome value, age, sex, BMI, duration of diabetes, insulin use, and antibiotic exposure. An AR(1) working correlation structure and robust sandwich standard errors were used. The month-12 SMI comparison was the primary time-specific contrast; all other comparisons were secondary. P values correspond to the adjusted month-12 contrasts. SMI, skeletal muscle index; SPPB, Short Physical Performance Battery; HbA1c, glycated hemoglobin A1c; HOMA-IR, homeostasis model assessment of insulin resistance; FPG, fasting plasma glucose.

### 3.3 Longitudinal changes in metabolic outcomes

In the adjusted GEE models, the group × time interactions were not significant for HbA1c (Wald χ² = 1.325, P = 0.857), HOMA-IR (Wald χ² = 4.245, P = 0.374), or FPG (Wald χ² = 8.743, P = 0.068), indicating no statistically significant evidence that their overall longitudinal trajectories differed between the intervention and control groups. The observed longitudinal trajectories with 95% CIs are presented in **Figure 3**. With the control group and month 1 as the reference categories, the group effects at month 1 were not significant for HbA1c (Wald χ² = 0.001, P = 0.977), HOMA-IR (Wald χ² = 3.098, P = 0.078), or FPG (Wald χ² = 1.803, P = 0.179). In the control group, the time effect was significant for HbA1c (Wald χ² = 20.900, P < 0.001), but not for HOMA-IR (P = 0.163) or FPG (P = 0.198). The corresponding baseline outcome value and age were significantly associated with all three metabolic outcomes (all P < 0.05), whereas sex, BMI, diabetes duration, insulin use, and antibiotic exposure were not significantly associated with these outcomes (**Table 4**). At month 12, no significant adjusted between-group difference was observed for HbA1c (adjusted between-group difference: 0.013%; 95% CI: −0.183 to 0.209; P = 0.894). The intervention group had lower adjusted HOMA-IR (adjusted between-group difference: −0.204; 95% CI: −0.371 to −0.038; P = 0.016) and FPG (adjusted between-group difference: −0.254 mmol/L; 95% CI: −0.473 to −0.034; P = 0.024) than the control group (**Table 3**).

**Figure 3:**
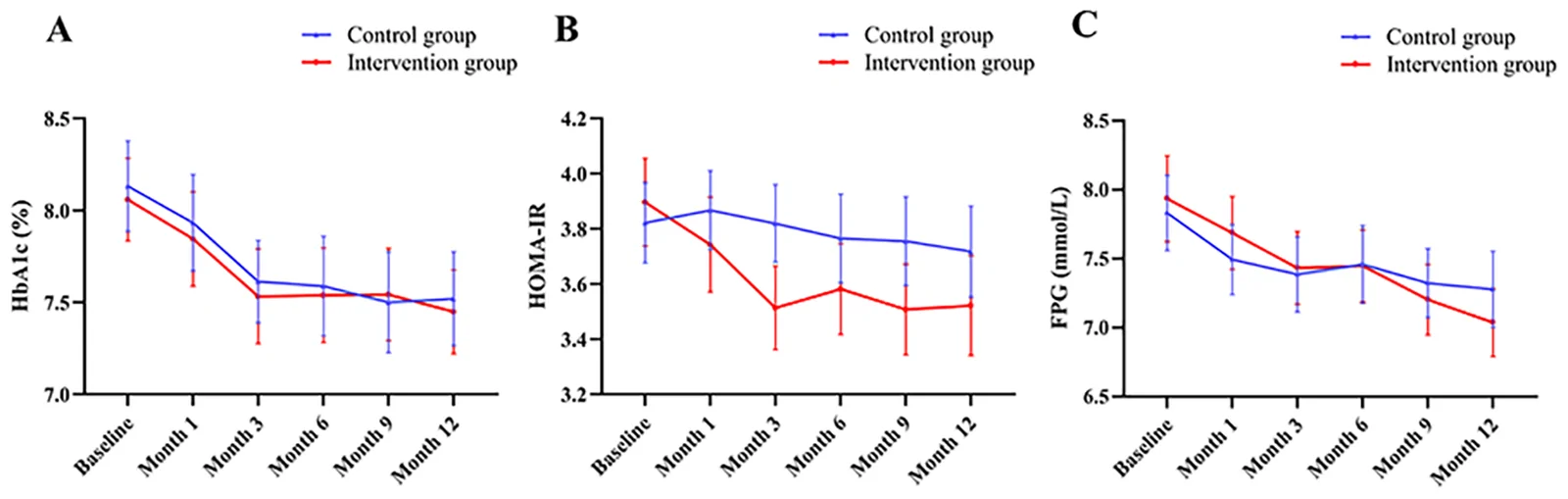
Longitudinal changes in HbA1c, HOMA-IR, and FPG. **(A)** HbA1c; **(B)** HOMA-IR; and **(C)** FPG. Values are presented as observed means with 95% CI at baseline and months 1, 3, 6, 9, and 12. At each assessment, the control and intervention groups included 63 and 65 participants, respectively. Adjusted GEE analyses showed no significant group × time interactions for HbA1c (P = 0.857), HOMA-IR (P = 0.374), or FPG (P = 0.068). CI, confidence interval; GEE, generalized estimating equation; HbA1c, glycated hemoglobin; HOMA-IR, homeostasis model assessment of insulin resistance; FPG, fasting plasma glucose.

**Table 4:** Longitudinal changes in HbA1c, HOMA-IR, and FPG.

Variable	HbA1c (%)			HOMA-IR			FPG (mmol/L)		
	Wald χ²	df	P	Wald χ²	df	P	Wald χ²	df	P
Group	0.001	1	0.977	3.098	1	0.078	1.803	1	0.179
Time	20.900	4	<0.001	6.530	4	0.163	6.019	4	0.198
Group × Time	1.325	4	0.857	4.245	4	0.374	8.743	4	0.068
Corresponding baseline outcome value	95.800	1	<0.001	69.200	1	<0.001	146.000	1	<0.001
Age	44.600	1	<0.001	57.300	1	<0.001	82.100	1	<0.001
Sex	0.089	1	0.765	0.018	1	0.895	0.001	1	0.980
BMI	0.056	1	0.812	0.189	1	0.663	0.442	1	0.506
Duration of diabetes	1.259	1	0.262	0.072	1	0.788	1.380	1	0.240
Insulin use	0.159	1	0.690	0.051	1	0.821	0.003	1	0.958
Antibiotic exposure	0.909	1	0.340	0.103	1	0.748	1.540	1	0.215

Values are Wald χ² statistics, degrees of freedom (df), and P values from GEE models. Measurements at months 1, 3, 6, 9, and 12 were modeled as repeated outcomes. Models included group, time, and group × time and were adjusted for the corresponding baseline outcome value, age, sex, BMI, diabetes duration, insulin use, and antibiotic exposure. An AR(1) correlation structure and robust sandwich standard errors were used. The group × time interaction represents differences in longitudinal trajectories between groups. Model-fit indices were: HbA1c, QIC/QICC = 289.106/295.381; HOMA-IR, 174.779/181.054; and FPG, 394.570/400.845. HbA1c, glycated hemoglobin A1c; HOMA-IR, homeostasis model assessment of insulin resistance; FPG, fasting plasma glucose; QIC, quasi-likelihood under the independence model criterion; QICC, corrected QIC.

### 3.4 Adverse events

During the 12-month follow-up, 24 participants (38.10%) in the control group and 29 (44.62%) in the intervention group experienced at least one adverse event. No significant between-group difference was detected in the overall incidence of adverse events (RR = 1.171, 95% CI: 0.773–1.774; P = 0.478). The corresponding total numbers of adverse events were 28 and 35, respectively. All recorded events were mild, and no serious adverse events or discontinuations due to adverse events were documented (**Table 5**).

**Table 5:** Adverse events in both groups during the 12-month follow-up.

Adverse events	Control group (n = 63)	Intervention group (n = 65)	Risk ratio (95% CI)	P
Hypoglycemia	3 (4.76%)	2 (3.08%)	—	—
Gastrointestinal discomfort	4 (6.35%)	5 (7.69%)	—	—
Abdominal bloating	3 (4.76%)	6 (9.23%)	—	—
Constipation	5 (7.94%)	6 (9.23%)	—	—
Loss of appetite	2 (3.17%)	2 (3.08%)	—	—
Increased appetite	3 (4.76%)	5 (7.69%)	—	—
Musculoskeletal injury	3 (4.76%)	5 (7.69%)	—	—
Fatigue or dizziness	5 (7.94%)	4 (6.15%)	—	—
Serious adverse events	0 (0.00%)	0 (0.00%)	—	—
Discontinuations due to adverse events	0 (0.00%)	0 (0.00%)	—	—
Total number of adverse events	28	35	—	—
Patients with ≥1 adverse event	24 (38.10%)	29 (44.62%)	1.171 (0.773–1.774)	0.478

Data are presented as n (%), except for total event counts. Participants with multiple events were counted once in the overall incidence but each event was included in the total count. The risk ratio compares the intervention group with the control group, and the P value was calculated using Fisher’s exact test. CI, confidence interval.

## 4 Discussion

Diabetic sarcopenia is an increasingly recognized complication of T2DM, characterized by progressive loss of skeletal muscle mass, muscle strength, and physical function (12, 13). However, conventional diabetes management strategies mainly emphasize glycemic control and cardiovascular risk reduction, often inadequately addressing muscle-related decline (7). In this study, exercise combined with dietary and microbiota-targeted care was associated with significantly different 12-month longitudinal trajectories of SMI, handgrip strength, gait speed, and SPPB score compared with conventional diabetes management alone, whereas no significant between-group differences were observed in the trajectories of HbA1c, HOMA-IR, or FPG. At month 12, the intervention group had significantly higher adjusted values for SMI, handgrip strength, gait speed, and SPPB score, and significantly lower adjusted values for HOMA-IR and FPG. No significant difference in adverse-event incidence was detected.

SMI, as a body size–adjusted indicator of muscle mass, is closely linked to metabolic health, physical independence, and long-term prognosis, and its deterioration reflects not only sarcopenia progression but also the cumulative burden of insulin resistance and chronic inflammation (27, 28). In this study, the intervention group showed more favorable longitudinal trajectories of SMI, handgrip strength, gait speed, and SPPB score. The significant group × time interaction for SMI indicated that the pattern of muscle-mass change over follow-up differed between groups. At month 12, the adjusted between-group difference in SMI was 0.282 kg/m², suggesting potentially relevant preservation or improvement of muscle mass that may help maintain physical function and independence in patients with established sarcopenia. These findings may reflect the ability of resistance-based exercise to stimulate muscle protein synthesis, improve neuromuscular recruitment, and counteract age- and diabetes-related anabolic resistance (29, 30). Previous clinical studies have similarly shown that exercise-based interventions generally produce greater improvements in muscle strength and physical performance than in muscle mass (23, 24). The concurrent differences in handgrip strength, gait speed, and SPPB score further support the functional relevance of the observed SMI difference, reflecting complementary aspects of physical performance, including strength, coordination, balance, and functional reserve (31, 32).

In addition, dietary counseling and microbiota-targeted care may have complemented the exercise program and contributed to the favorable longitudinal trajectories of SMI, handgrip strength, gait speed, and SPPB score observed in the intervention group. This possibility is consistent with the concept of a gut–muscle axis, through which dietary and microbiota-related factors may influence skeletal muscle health. Potential pathways include modulation of microbial metabolite production, intestinal barrier integrity, systemic inflammation, and insulin sensitivity (15–18). Prior trials have shown improvements in strength and function with exercise alone, but effects on SMI are frequently attenuated in older individuals with long-standing metabolic dysfunction (33, 34). Compared with previous studies focusing solely on exercise interventions, which often report modest or inconsistent effects on muscle mass in patients with T2DM (35), the present study provides real-world observational evidence that a multifaceted care package involving exercise, dietary counseling, and optional microbiota-targeted supplementation was associated with favorable differences in both muscle mass and physical function.

With respect to metabolic outcomes, the group × time interactions were not significant for HbA1c, HOMA-IR, or FPG, providing no evidence of different longitudinal trajectories between groups. The comparable HbA1c findings may reflect the conventional diabetes management received by both groups and do not support superior overall glycemic control with the combined care package. Although HOMA-IR and FPG were lower in the intervention group at month 12, these secondary time-specific findings should be interpreted cautiously because the overall interactions were nonsignificant. This dissociation between HbA1c and insulin resistance highlights the multifaceted nature of metabolic regulation in patients with diabetic sarcopenia (12, 13). The month-12 differences in HOMA-IR and FPG could potentially be related to exercise-associated changes in skeletal-muscle insulin sensitivity, dietary changes, concurrent clinical management, or residual confounding (36, 37).

Skeletal muscle is the primary site of insulin-stimulated glucose uptake, and greater muscle mass and function may contribute to whole-body insulin sensitivity (38). In parallel, dietary modification and microbiota-targeted interventions have been proposed to influence metabolic endotoxemia and hepatic and peripheral insulin signaling (39). The significant reduction in HOMA-IR and FPG further supports the notion that sustained lifestyle-based interventions can exert cumulative metabolic benefits beyond short-term glycemic fluctuations (40). Previous studies evaluating dietary modulation or probiotic supplementation alone reported variable effects on insulin resistance and fasting glucose, often limited by short follow-up durations (41). Our findings suggest that combining these approaches with structured exercise may produce additive or synergistic metabolic effects, particularly in insulin resistance, which is a central driver of both poor glycemic control and muscle catabolism in T2DM (42). However, these findings should be interpreted cautiously because the overall group × time interactions for HbA1c, HOMA-IR, and FPG were not statistically significant and therefore do not support a general claim of superior metabolic benefit. Moreover, without microbiota, metabolite, endotoxemia, or signaling measurements, microbiota modification and related mechanisms remain unconfirmed hypotheses.

Baseline outcome values were significantly associated with their corresponding longitudinal measures, indicating that initial muscle and metabolic status remained important determinants of subsequent outcomes (43). Age was also associated with handgrip strength, gait speed, SPPB score, and all three metabolic outcomes, consistent with age-related declines in neuromuscular function, mobility, metabolic regulation, and insulin sensitivity. These findings highlight the importance of considering both baseline status and age when interpreting longitudinal changes and developing individualized management strategies for patients with diabetic sarcopenia (7, 13, 19).

Importantly, the overall incidence and spectrum of adverse events were comparable between the intervention and control groups, and no serious adverse events or discontinuations due to adverse events were documented. All reported events were mild and self-limited, including gastrointestinal discomfort and transient musculoskeletal symptoms, which are commonly encountered in lifestyle-based interventions (44). These findings provide preliminary evidence regarding the safety and feasibility of combined exercise and microbiota-targeted care in patients with diabetic sarcopenia. Given concerns that older patients with diabetes may be at increased risk of hypoglycemia or exercise-related injury, the absence of a higher adverse event burden in the intervention group is clinically reassuring (45, 46). Together with the observed muscle-related differences, these findings indicate the potential clinical relevance of this integrated management approach, which warrants further evaluation in prospective studies of patients with diabetic sarcopenia.

However, several limitations of this study should be acknowledged. First, the retrospective, non-randomized design was subject to selection bias, confounding by indication, and residual confounding. Treatment decisions may have been influenced by patients’ baseline health status, functional capacity, motivation, dietary habits, anticipated ability to participate, and physicians’ judgment. Although the GEE models were adjusted for the corresponding baseline outcome value, age, sex, BMI, diabetes duration, insulin use, and antibiotic exposure, unmeasured differences in physical activity, dietary and protein intake, comorbidities, renal function, diabetic complications, frailty, sarcopenia severity, detailed antidiabetic medication regimens, and treatment changes may have affected the observed associations. Baseline comparability assessed using standardized differences and multivariable adjustment cannot eliminate these sources of confounding. Second, the final analysis included only patients with complete measurements at all six assessment points and, in the intervention group, those meeting the prespecified adherence criteria for the additional 8-week intervention. Because adherence was assessed after treatment initiation, selection was partly based on a post-exposure characteristic potentially related to health status, motivation, tolerability, early response, or adverse events. Adherence was assessed retrospectively from routine clinical records, and the 80% threshold was pragmatic rather than biologically validated, creating the potential for incomplete documentation and misclassification. A complete longitudinal analysis based on the treatment strategy initiated at baseline could not be performed because baseline information, repeated outcome measurements, reasons for inadequate adherence, discontinuation or treatment modification, and safety data were not sufficiently complete or consistent for excluded patients. This adherent complete-case selection may have introduced bias, overestimated favorable associations, underestimated harms, and limited the generalizability of the findings to patients with lower adherence or incomplete follow-up. Third, the intervention was a heterogeneous care package consisting of structured exercise, individualized dietary counseling, and optional microbiota-targeted supplementation prescribed at the treating physician’s discretion. The type, formulation, dose, and frequency of probiotic, prebiotic, and synbiotic supplementation varied among patients. The intervention group also received more supervised care and professional contact than the control group. Therefore, the influence of differential clinical contact and the individual contributions of exercise, dietary counseling, and supplementation could not be separated. Gut microbiota composition, microbial metabolites, inflammatory biomarkers, and muscle-quality measures were not assessed. Consequently, the study could not determine whether the intervention altered the gut microbiota, identify microbiota-mediated mechanisms, or demonstrate additive or synergistic effects. Fourth, the supervised exercise program and prescribed supplementation lasted only 8 weeks, and no standardized maintenance program was implemented. Subsequent home exercise, dietary adherence, supplement use, medication changes, and other co-interventions were not consistently documented. Therefore, the differences observed at month 12 cannot be attributed exclusively to the initial intervention or interpreted as evidence of a durable intervention effect. Fifth, outcome assessors were not specifically blinded to treatment status, and formal intra-rater and inter-rater reliability data were unavailable. Measurement bias therefore cannot be excluded, particularly for handgrip strength and physical-performance outcomes. Multiple secondary outcomes and time-specific contrasts were also examined, increasing the risk of chance findings due to multiple testing. Finally, the single-center setting and moderate sample size further limit generalizability and the ability to assess uncommon adverse events or hard clinical outcomes. Future prospective multicenter studies should use randomized designs, standardized intervention components, objective adherence monitoring, blinded outcome assessment, and prespecified analyses. Direct measurements of gut microbiota, microbial metabolites, inflammatory biomarkers, and muscle quality are needed to clarify potential mechanisms and distinguish the contributions of individual intervention components. Longer follow-up should also assess intervention maintenance, adverse events, and clinically important outcomes.

## 5 Conclusion

In this retrospective cohort study, structured exercise combined with dietary counseling and optional microbiota-targeted supplementation was associated with more favorable longitudinal trajectories of SMI, muscle strength, and physical performance than conventional diabetes management alone. No between-group difference in HbA1c trajectory was observed, and the metabolic findings were less consistent. No statistically significant difference in adverse-event incidence was detected in this sample. However, the retrospective design, residual confounding, post-initiation selection, and intervention heterogeneity preclude causal inference or separation of individual component effects. Future prospective, multicenter randomized trials incorporating standardized interventions and mechanistic assessments are warranted to confirm these findings and guide clinical implementation.

## Data Availability

The original contributions presented in the study are included in the article/**Supplementary Material**. Further inquiries can be directed to the corresponding author.
