# Supplementary material for: Longitudinal outcomes associated with structured exercise and dietary and microbiota-targeted care in patients with diabetic sarcopenia: a retrospective cohort study
Source: Front Endocrinol (Lausanne). 2026 Sep 22;17:1923121. doi: 10.3389/fendo.2026.1923121 (PMC13638599; doi:10.3389/fendo.2026.1923121)
Supplement: Supplementary file 1 [file Table1.doc]

**Table S1.** Components of dietary counseling and microbiota-targeted supplementation in the intervention group.

| Intervention component | Main formulation/components | Dose and frequency | Duration | Patients, n (%) |
| --- | --- | --- | --- | --- |
| Structured dietary counseling | Individualized fiber-rich dietary recommendations | Delivered at baseline and reinforced during follow-up | 8 weeks | 65 (100.0) |
| Probiotic supplementation | Preparations containing viable Bifidobacterium, Lactobacillus, and/or Enterococcus species | Product-specific prescribed dose, generally administered orally twice daily | 8 weeks | 30 (46.2) |
| Prebiotic supplementation | Inulin and/or fructo-oligosaccharides | Total daily dose of approximately 5–10 g | 8 weeks | 18 (27.7) |
| Synbiotic supplementation | Bifidobacterium and/or Lactobacillus species combined with inulin or fructo-oligosaccharides | Product-specific prescribed dose, generally administered orally once daily | 8 weeks | 22 (33.8) |
| Dietary counseling without supplementation | No microbiota-targeted supplementation | — | 8 weeks | 12 (18.5) |
